# Anti-LRP/LR Specific Antibody IgG1-iS18 Impedes Adhesion and Invasion of Liver Cancer Cells

**DOI:** 10.1371/journal.pone.0096268

**Published:** 2014-05-05

**Authors:** Carryn Chetty, Thandokuhle Khumalo, Bianca Da Costa Dias, Uwe Reusch, Stefan Knackmuss, Melvyn Little, Stefan F. T. Weiss

**Affiliations:** 1 School of Molecular and Cell Biology, University of the Witwatersrand, Johannesburg, Gauteng, The Republic of South Africa (RSA); 2 Affimed Therapeutics AG, Technologiepark, Im Neuenheimer Feld, Heidelberg, Baden-Wuerttemberg, Germany; The Scripps Research Institute Scripps Florida, United States of America

## Abstract

Two key events, namely adhesion and invasion, are pivotal to the occurrence of metastasis. Importantly, the 37 kDa/67 kDa laminin receptor (LRP/LR) has been implicated in enhancing these two events thus facilitating cancer progression. In the current study, the role of LRP/LR in the adhesion and invasion of liver cancer (HUH-7) and leukaemia (K562) cells was investigated. Flow cytometry revealed that the HUH-7 cells displayed significantly higher cell surface LRP/LR levels compared to the poorly-invasive breast cancer (MCF-7) control cells, whilst the K562 cells displayed significantly lower cell surface LRP/LR levels in comparison to the MCF-7 control cells. However, Western blotting and densitometric analysis revealed that all three tumorigenic cell lines did not differ significantly with regards to total LRP/LR levels. Furthermore, treatment of liver cancer cells with anti-LRP/LR specific antibody IgG1-iS18 (0.2 mg/ml) significantly reduced the adhesive potential of cells to laminin-1 and the invasive potential of cells through the ECM-like Matrigel, whilst leukaemia cells showed no significant differences in both instances. Additionally, Pearson's correlation coefficients suggested direct proportionality between cell surface LRP/LR levels and the adhesive and invasive potential of liver cancer and leukaemia cells. These findings suggest the potential use of anti-LRP/LR specific antibody IgG1-iS18 as an alternative therapeutic tool for metastatic liver cancer through impediment of the LRP/LR- laminin-1 interaction.

## Introduction

Cancer is a global burden that has been shown to be the leading cause of death in economically developed countries and the second leading cause of death in economically developing countries[1]. According to the World Cancer Research Fund (WCRF), an estimated 14.1 million cases of cancer were diagnosed in the year 2012 and it is predicted that approximately 24 million new cases of cancer will be diagnosed by the year 2035, globally (http://www.wcrf.org/cancer_statistics/). Currently, lung cancer has been identified as the most commonly diagnosed cancer type, with the two cancer types central to the present study namely liver cancer and leukaemia, being ranked as sixth and eleventh most diagnosed cancer types, respectively (GLOBOCAN). It has been reported that approximately 782000 cases of liver cancer and 352000 cases of leukaemia were diagnosed in the year 2012 (http://www.wcrf.org/cancerstatistics/world cancer statistics.php), thus indicating the pressing need to develop effective treatments against cancer.

Cells are largely dependent on the extracellular matrix (ECM), which is the non-cellular component of all tissues and organs that provides a physical scaffold to cellular components and also assists with initiation of essential biochemical processes needed for proper tissue differentiation, homeostasis and morphogenesis[2]. Cells adhere to the ECM via the action of ECM receptors[2]. Particularly, the non-integrin 37-kDa/67-kDa laminin receptor (LRP/LR) is a major component of the extracellular matrix, assisting in numerous physiological processes[3], [4], [5]. It is suggested that 37-kDa LRP is the precursor of the 67-kDa high affinity laminin receptor LR, however, the exact mechanism by which the precursor forms the receptor is unknown[6].

LRP/LR is predominantly a transmembrane receptor, however, it is also evident in the nucleus and the cytosol[7], [8]. In the nucleus, LRP/LR plays a critical role in the maintenance of nuclear structures whilst in the cytosol, it assists in translational processes[8]. As a transmembrane receptor, LRP/LR serves several functions such as cell migration[9], cell-matrix adhesion[10], cell viability and proliferation[3], [4], [5].

LRP/LR has been shown to have a high binding affinity for laminin-1. Laminin-1 is part of a family of laminins, which are extracellular matrix proteins that constitute several non-collagenous glycoproteins that are found in the basement membrane[11], [12]. This glycoprotein is believed to play critical roles in cell attachment[11], assembly of the basement membrane[11], cell growth and differentiation[13], cell migration[11], [14], neurite outgrowth[11], [15] and angiogenesis[16]. Laminin-1 has also been shown to promote the invasive phenotype of tumorigenic cells[17].

LRP/LR has been found to be over-expressed on the surface of several tumorigenic cells[18]. The result of this over-expression is an increased interaction between LRP/LR and laminin-1, and this interaction has been shown to be crucial in enhancing adhesion and invasion – two key components of metastasis[19]. Essentially, laminin-1 in the basement membrane interacts with LRP/LR on the surface of tumorigenic cells leading to adhesion[19]. This, in turn, results in the secretion of proteolytic enzymes such as type IV collagenase in order to hydrolyse type IV collagen in the basement membrane, thereby allowing tumorigenic cells to invade and eventually translocate to a secondary site[19].

Since the LRP/LR-laminin-1 interaction has been identified as the crucial event in adhesion and invasion, blockage of this interaction could be deemed as an essential mechanism to treat metastatic cancer. This implicates LRP/LR as a target for the treatment of metastatic cancer. Furthermore, several studies have shown that application of anti-LRP/LR specific antibodies significantly reduces the adhesive and invasive potential of certain tumorigenic cells, such as HT1080 fibrosarcoma[18], lung[4], cervical[4], colon[4], prostate[4], breast[20] and oesophageal[20] cancer cells. Particularly, anti-LRP/LR specific antibody IgG1-iS18 has been suggested to interrupt the LRP/LR-laminin-1 interaction [4], thus IgG1-iS18 may be deemed as a possible therapeutic tool in the treatment of metastatic cancer.

In this study, the ability of anti-LRP/LR-specific antibody IgG1-iS18 to impede the adhesive and invasive potential of leukaemia and liver cancer cells was investigated. Due to the high incidence and mortality rates regarding these two cancer types, alternative therapeutic options become a necessity. It is noteworthy that similar studies have been conducted, however, it is possible that not all metastatic cancer cell types may be responsive to IgG1-iS18 treatments. It therefore becomes necessary to carry out these metastatic studies on different cancer types in order to gain insight into the use of the antibody as an alternative broad spectrum therapeutic antibody for the treatment of various cancer types. Thus, this study was conducted with the aim of determining whether IgG1-iS18 is capable of significantly reducing the adhesive and invasive potential of leukaemia and liver cancer cells, therefore providing the possibility for the antibody to be used as an alternative therapeutic tool in the treatment of these two cancer types.

## Materials and Methods

### Cell culture and conditions

Human breast adenocarcinoma (MCF-7), liver carcinoma (HUH7) and leukaemia (K562) cell lines obtained from ATCC were cultured in Dulbecco's modified Eagle's medium (DMEM) high glucose (4.5 g/l) supplemented with 10% fetal calf serum and 1% penicillin/streptomycin at 5% CO_2_ and 37°C.

### Reagents and antibodies

Matrigel used for cell invasion assays is derived from the Engelbreth-Holm-Swarm (EHS) mouse sarcoma and was obtained from BD Biosciences.

Laminin-1used for cell adhesion assays was obtained from Sigma-Aldrich.

Chloramphenicol acetyl transferase (CAT) antibody was obtained from Sigma-Aldrich.

IgG1-iS18 was recombinantly produced in a mammalian expression system as reported by

Zuber et al., (2008)

### Confocal microscopy

In order to visualize the location of LRP/LR on the cell surface, confocal microscopy was employed. Cells were first seeded on coverslips and allowed to reach 70% confluency. Cells were fixed in 4% paraformaldehyde in PBS for approximately 15 minutes followed by several washes with PBS. Cells were blocked in 0.5% BSA in PBS for 5-10 minutes. After one PBS wash, excess PBS was blotted off. Cover slips containing cells were placed on a glass slide (with cells facing upwards) and this was followed by addition of primary antibody IgG1-iS18 (1∶100) diluted in 0.5% BSA. Post an overnight incubation at 4°C, coverslips were rinsed thrice in PBS/BSA. After addition of the FITC-coupled secondary antibody that had been diluted in 0.5% BSA, incubation in the dark was allowed for 1 hour. Followed by three washes as before, DAPI diluted in PBS was then administered for 5–10 minutes to allow for staining of the nucleus. Cells were finally washed once in PBS alone and mounted onto a clean slide using GelMount (Sigma-Aldrich). A period of 45 minutes was allocated to allow for setting to take place.

### Flow cytometry

Quantification of cell surface levels of LRP/LR was conducted using flow cytometry. EDTA(5 mM) in PBS was used to facilitate detachment of adherent cells which was followed by centrifugation at 1200 rpm, 10 min. Cells were subsequently fixed by re-suspending cells in PFA for 10 min at 4°C. Cells were again centrifuged in 1X PBS which allowed for the preparation of five cell suspensions, one to which no antibody was added (thus serving as the unstained control), one to which anti-CAT antibody was added (serving as an isotype control) and one to which anti-LRP/LR specific antibody IgG1-iS18 was added. The remaining two cell suspensions were incubated only in PBS in order to be used as negative controls for the IgG1-iS18 and anti-CAT antibody. All suspensions were incubated at room temperature for 1 hour. Following three washing steps with 1X PBS, goat anti-human phycoerythrin (PE)-coupled secondary antibody (Beckman Coulter) was added to the cell suspension containing the IgG1-iS18 primary antibody as well as one of the suspensions that was incubated in PBS only. The cell suspension that was incubated with the anti-CAT antibody as well as the remaining cell suspension that was incubated only in PBS, were both supplemented with a goat anti-rabbit allophycocyanin (APC)-coupled secondary antibody followed by another 1 hour incubation period of all cell suspensions. Furthermore, three post-incubation washes were performed and cell suspensions were analysed using the BD Accuri C6 flow cytometer. Experiments were performed in triplicate and repeated at least three times.

### SDS PAGE and Western blotting

Total LRP/LR levels were determined by the use of sodium dodecyl sulphate polyacrylamide gel electrophoresis (SDS-PAGE). To perform the SDS-PAGE, 10 µg of total protein was used. Proteins that were separated according to size by SDS-PAGE were then identified by application of specific antibodies in the process of Western blotting. The proteins resolved on the polyacrylamide gel were transferred onto a polyvinylidene fluoride (PVDF) membrane using 1X transfer buffer (20% methanol in 192 mM glycine and 25 mM Tris) for 45 minutes at 350 mV and a semi-dry transferring apparatus. Blocking buffer (3% BSA in 1X PBS Tween) was then used in order to block the blotted membrane for 1 hour. Once blocked, the membrane was probed with anti-LRP/LR specific primary antibody IgG1-iS18 (1∶10000) for 1 hour. Prior to incubation of the membrane with goat-anti-human-peroxidase (1∶5000) secondary antibody, three washes with 1X PBS Tween were performed. A further three washes in 1X PBS Tween were performed after incubation in the secondary antibody, followed by the detection of HRP by use of an enhanced chemiluminescent substrate (Thermo scientific). The resulting fluorescence was developed and fixed onto an X-ray film. Experiments were executed in triplicate and repeated at least 3 times.

### Adhesion assay

In order to assess the adhesive potential of the varying tumorigenic cell lines to the basement membrane *in vitro*, laminin-1 (10 µg/ml)(BD Biosciences) was used to coat 96 microwell plates, leaving uncoated wells to be used as negative controls. After the coating of the wells for 1 hour and washing with 0.1% BSA in DMEM, other protein binding sites on the microwell plate were blocked using 100 µl of 0.5% BSA in DMEM for one hour. Cells were suspended in serum-free culture medium and added to wells at a density of 4×10^5^ cells/ml in order to assess the adhesion potential. Furthermore, cells that have been pre-incubated with IgG1-iS18 (0.2 µg/ml) and with anti-CAT (Sigma, 0.2 µg/ml) antibody as the negative control were added to the relevant wells in order to examine the effects of the antibodies on the adhesion potential of the cells. The plates were incubated at 37°C for 1 hour and thereafter, non-adherent cells were washed away with PBS and adherent cells were fixed with 4% paraformaldehyde for 10 minutes. Adherent cells were stained with 0.1% crystal violet. The stain was extracted using 1% SDS and the absorbance of the extracted sample at 550 nm was assayed as a measure of the adhesive potential. Experiments were performed in triplicate and repeated at least three times.

### Invasion assay

*In vitro* analysis of the ability of the tumorigenic cell lines to invade the basement membrane was carried out using the ECM-like Matrigel. Serum-free cold culture medium (DMEM) was used in order to dilute the Matrigel and this diluted gel was dispensed onto the upper chamber of a 24 transwell plate (BD falcon, 8 µm pore size). This gel was then allowed to solidify for approximately 5 hours at 37°C. After being harvested, cells were resuspended in serum-free culture media at a density of 1×10^6^ cells/ml. Antibody treatments required cells to be incubated with IgG1-iS18 (0.2 µg/ml) or the negative control anti-CAT (Sigma, 0.2 µg/ml) antibody. Cells were subsequently loaded onto the upper Matrigel-covered chamber and incubated for 18 hours. The lower chamber was filled with 500 µl of culture media containing 10% FCS (for the test) and no FCS (for the control), and incubated at 37°C for 18 hours. This was followed by aspiration of the media in the lower and upper chamber. Non-invasive cells were then removed by use of a cotton swab. The remaining invasive cells were then washed with 300 µl of PBS and fixed using 300 µl of 4% paraformaldehyde, 10 min. Cells were stained using 0.5% toluidine blue dye and after extraction of the dye using 1% SDS, absorbance was then measured at 620 nm using an ELISA reader. Experiments were carried out in triplicate and repeated at least three times.

### Statistical evaluations

The two-tailed Student's t-test with a confidence interval of 95% was used in order to analyse the data, with p-values of less than 0.05 being considered significant. The extent or degree of association between LRP/LR levels on the cell surface and invasive/adhesive potential was measured using the Pearson's correlation coefficient. The Pearson's correlation coefficient was also used to measure the correlation between the adhesive and invasive potential of the cell lines. A positive coefficient was an indication of direct proportionality between the two variables, whereas a negative coefficient implied inverse proportionality.

## Results

### Liver cancer and leukaemia cells reveal LRP/LR on the cell surface

**Figure 1:**
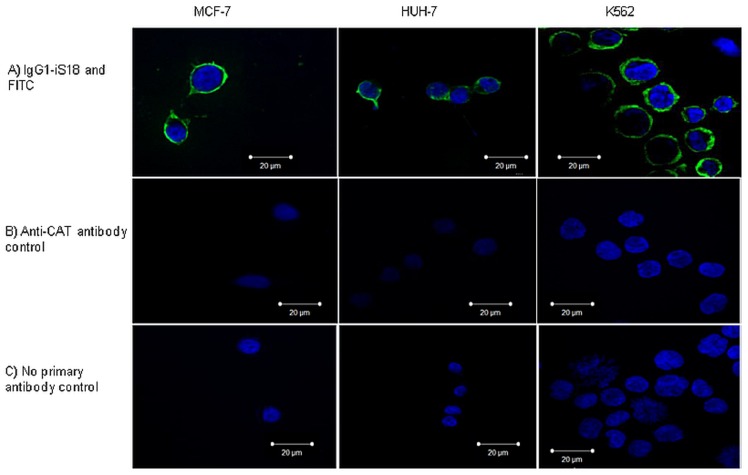
Visualisation of LRP/LR on the surface of liver cancer (HUH-7) and leukaemia (K562) cells. Cells were non-permeabilized in order to allow for visualisation of the cell surface. a) Cells were labelled with primary antibody IgG1-iS18 and a FITC-coupled secondary antibody. b) Cells were labelled with anti-chloramphenicol acteyltranferase (CAT) antibody as the negative control. c) Cells were labelled only with the FITC-coupled secondary antibody to confirm that the secondary antibody does not bind non-specifically.

Pivotal to the occurrence of metastasis is the interaction between laminin-1 and LRP/LR on the cell surface. Hence, it was necessary to visualize cell surface LRP/LR as a means of confirmation that cells indeed do display LRP/LR on their surface. Both tumorigenic cell lines, as well as the poorly-invasive breast cancer control, revealed LRP/LR on the cell surface as depicted in Fig.1 a). The green fluorescence in the images below is indicative of cell surface LRP/LR as cells were non-permeabilized and the secondary antibody was shown to not bind non-specifically, as confirmed by the controls depicted in Fig.1 b) and Fig.1 c) below. Anti-CAT antibody was used as a negative control due to its ability to bind specifically to chloramphenicol acetyltransferase (CAT) which is a bacterial protein and is therefore absent in mammalian cells.

### High percentages of tumorigenic cells display LRP/LR on the cell surface

Although confocal microscopy confirmed that the cell lines do indeed display LRP/LR on their cell surface, further quantification of the cell surface levels of LRP/LR was required. Flow cytometry was employed for this quantification.

**Figure 2:**
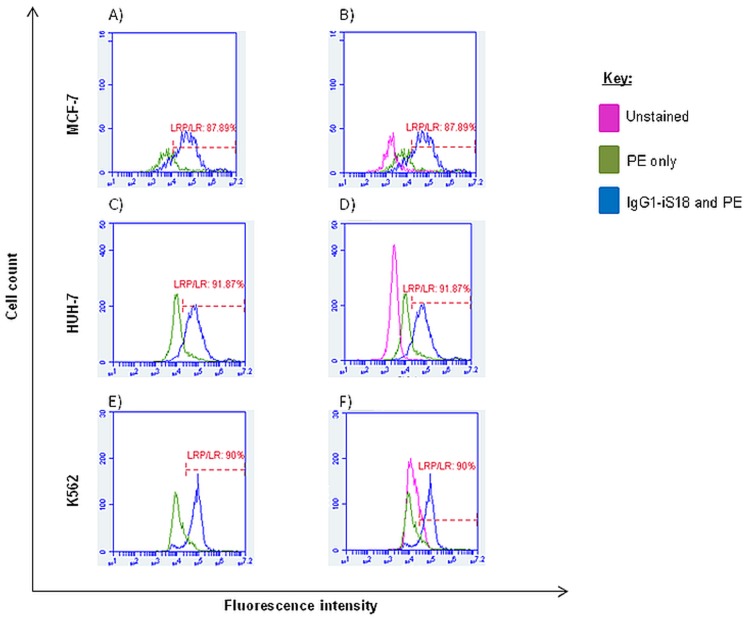
Quantification of liver cancer (HUH-7) and leukaemia (K562) cells within a population which exhibit LRP/LR on their cell surface. The first peak in graphs A, C and E is representative of non-labelled cells i.e. cells labelled with goat anti-human PE-coupled secondary antibody only, whereas the second peak is indicative of cells labelled with both anti-LRP/LR specific antibody IgG1-iS18 and the secondary antibody, both at a concentration of 30 µg/ml. Graphs B, D and F depict the inclusion of an unstained control to show no non-specific secondary antibody binding. Experiments were carried out in triplicate and repeated at least three times with 20 000 cells being counted per sample.

As shown in Fig.2 A, C and E, all three tumorigenic cell lines revealed high percentages of cells within a specific population that display LRP/LR on the cell surface, with the shift between the two peaks in each graph being indicative of a change in fluorescence intensity due to the cell-surface staining of the cells with anti-LRP/LR specific antibody IgG1-iS18 and the fluorochrome-coupled secondary antibody. HUH-7 liver cancer cells displayed a higher percentage of cells exhibiting LRP/LR on the cell surface in comparison to K562 leukaemia cells as well as the poorly-invasive breast cancer (MCF-7) control cell line. Fig. 2 B, D and F additionally include the unstained control and this control served to show that the PE secondary antibody does not bind non-specifically. The shifts in fluorescence intensity of unstained, APC only and anti-CAT-APC labelled cells (the negative controls) are represented in the Fig S1. It is also noteworthy to add that cell debris and cell aggregates were excluded from analysis as they were outside the defined gate (Fig. S3).

### Liver cancer cells display significantly higher and leukaemia cells display significantly lower cell surface LRP/LR levels compared to poorly-invasive breast cancer cells

**Figure 3:**
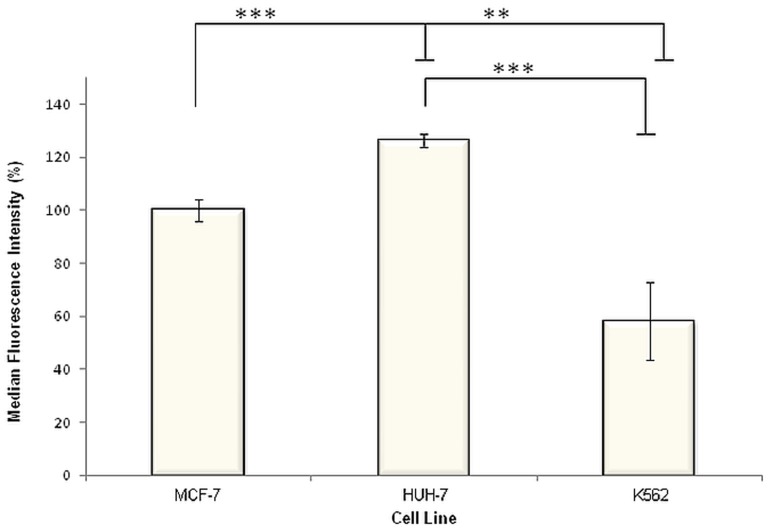
Quantification of cell surface LRP/LR levels on liver cancer (HUH-7) and leukaemia (K562) cells by flow cytometry. Cells were labelled with primary antibody IgG1-iS18 (1∶25) and anti-human phycoerythrin (PE) secondary antibody. 20000 cells were analyzed across all three cell lines, and the median fluorescence intensities (MFI) were used as an indicator of cell surface LRP/LR levels. The MFI values indicated in the last column of Table 1 were used in order to construct this figure and it is noteworthy that the MFI value corresponding to the MCF-7 cell line was set to 100%. Experiments were performed in triplicate and repeated at least three times. **p = 0.0025, ***p<0.0001.

In addition to the percentage of cells exhibiting LRP/LR on their cell surface, the actual cell surface LRP/LR levels within a specific cell population was analyzed using flow cytometry. The same number of cells (20000 cells) within specific populations of the three tumorigenic cell lines were labelled with the same concentration (30 µg/ml) of previously-mentioned primary and secondary antibodies over the same time period. Thus, the more LRP/LR that is present on the surface of the tumorigenic cells, the more primary antibody IgG1-iS18 would bind to LRP/LR and subsequently, the more IgG-specifc fluorochrome-coupled secondary antibody would bind to the primary antibody. Thus, the median fluorescence intensities (MFI) would differ between the three cell lines (Table 1) and therefore can be used as an indicator of cell surface LRP/LR levels. It was observed that, in comparison to the poorly-invasive MCF-7 breast cancer control cell line, liver cancer cells (HUH-7) displayed higher levels of LRP/LR on their cell surface (Fig.3). Additionally, K562 leukaemia cells revealed lower cell surface LRP/LR levels in comparison to the MCF-7 cells (Fig.3).

**Table 1:** Median fluorescence intensity (MFI) values as an indicator of differential expression of LRP/LR between MCF-7, HUH-7 and K562 cell lines.

Cell lines	MFI of unstained cells	MFI of cells labelled with IgG1-iS18 and PE	(MFI of cells labelled with IgG1-iS18 and PE) – (MFI of unstained cells)
MCF-7	1623.666667	38753.16667	37129.5
HUH-7	2678.166667	49556	46877.83333
K562	13823.75	35392	21568.25

*All values are representative of an average of results obtained from experiments that were performed in triplicate and repeated three times.

The median fluorescence intensities obtained post anti-CAT labelling and detection demonstrate that there is no significant difference in the degree of cell-surface CAT staining across all three cell lines (Fig. S2)

### Total LRP/LR levels do not differ significantly between the tumorigenic cell lines

**Figure 4:**
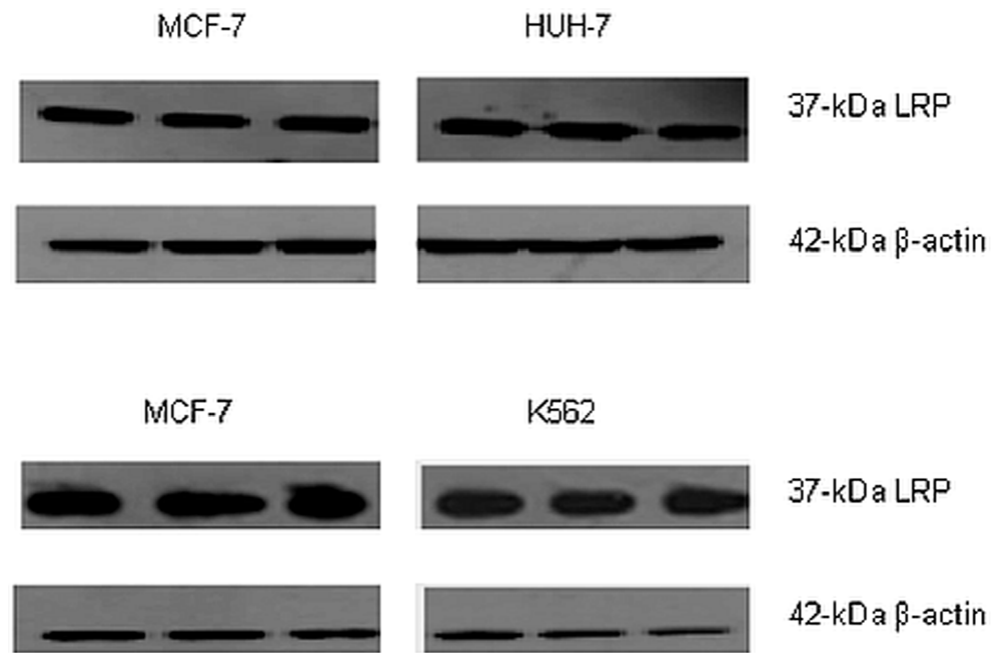
Detection of the relative expression of total 37(HUH-7) and leukaemia (K562) cell lines. Anti-LRP/LR specific antibody IgG1-iS18 was used as the primary antibody in conjunction with a secondary HRP-coupled antibody. β-actin was employed as a loading control.

As previously mentioned, LRP/LR does not exclusively occur on the cell surface but is additionally seen in the nucleus and cytosol, hence Western blot analysis was performed in order to assess total LRP/LR levels. Experiments were performed in triplicate and repeated three times. A representative blot is depicted in Fig.4. It is noteworthy to state that only the 37 kDa laminin receptor precursor could be detected by use of anti-LRP/LR specific antibody IgG1-iS18.

**Figure 5:**
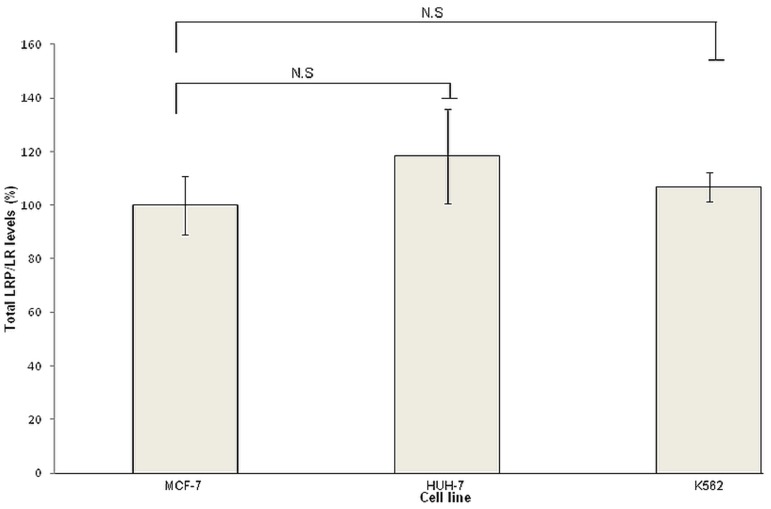
Total LRP levels of liver cancer (HUH-7) and leukaemia (K562) cell lines detected by Western blot analysis using primary anti-LRP/LR specific antibody IgG1-iS18 and goat anti-human HRP secondary antibody. Quantification was conducted using densitometry and data are representative of experiments carried out in triplicate and repeated three times. Non-significant (NS): p>0.05.

Following detection of LRP, quantification of total LRP levels was required and densitometry was employed to achieve this. Fig.5 depicts the densitometric analysis, which revealed that statistically, there was no significant difference observed in total LRP levels between the three tumorigenic cell lines.

### IgG1-iS18 significantly impedes the adhesive potential of liver cancer cells

Pivotal to the initiation of invasion is the adhesion of a tumorigenic cell to the basement membrane through the LRP/LR-laminin-1 interaction as it allows for other interactions to occur that facilitate degradation of the basement membrane. Cells were incubated with IgG1-iS18 and anti-CAT antibodies (0.2 mg/ml) and after an hour, absorbance readings of the resultant solution were indicative of the degree of cell attachment to the laminin-1-coated plates.

**Figure 6:**
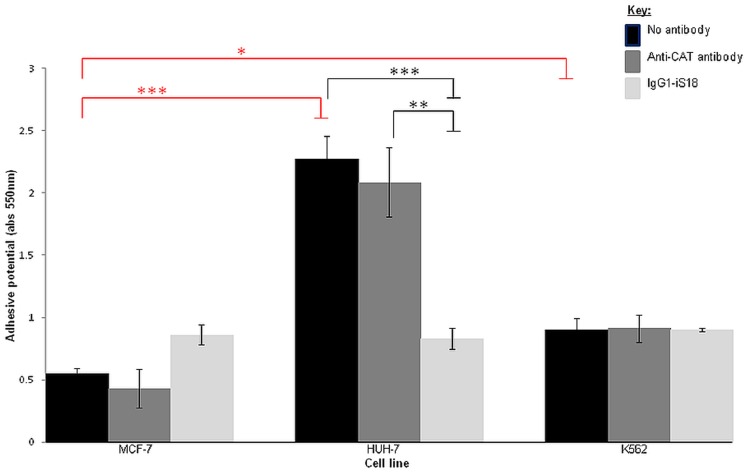
Effect of anti-LRP/LR specific antibody IgG1-iS18 on the adhesion of liver cancer (HUH-7) and leukaemia (K562) cells to laminin-1 determined through adhesion assays. p-values shown in red (*p = 0.0238; ***p = 0.0002) are indicative of the increase in adhesive potential of liver cancer (HUH-7) and leukaemia (K562) cells in comparison to the breast cancer (MCF-7) control cell line. p-values shown in black (**p = 0.0031; ***p = 0.0005) represent the reduction in adhesive potential after treatment of cells with appropriate antibodies. A reduction of 63.35% in the adhesive potential was observed upon administration of IgG1-iS18 to the HUH-7 liver cancer cells. Data represents experiments performed in triplicate and repeated at least three times.

As depicted in Fig.6, the no antibody control allowed for the determination of the adhesive potential of the cell lines and it was observed that both liver cancer (HUH-7) as well as leukaemia cells (K562) were more adherent than the poorly-invasive breast cancer (MCF-7) control cells. However, IgG1-iS18 was only effective at impeding the adhesive potential of liver cancer cells and no significant reduction in adhesion was observed for leukaemia cells treated with the anti-LRP/LR specific antibody IgG1-iS18. As expected, the anti-CAT control antibody did not have a significant effect on the adhesive potential of the tumorigenic cell lines.

### Invasion of the Matrigel by liver cancer cells (HUH-7) is significantly impeded by anti-LRP/LR specific antibody IgG1-iS18

Invasion of the basement membrane is considered as a pre-requisite for the progression of a metastatic cancer, hence invasion assays using a Matrigel, which mimics the components of the basement membrane, were performed to determine the invasive potential of the tumorigenic cell lines. Similarly to the adhesion assays, the no antibody control allowed for the determination of the invasive potential of the cell lines. Furthermore, cells were treated with anti-LRP/LR specific antibody IgG1-iS18 and anti-CAT antibody (0.2 mg/ml).

**Figure 7:**
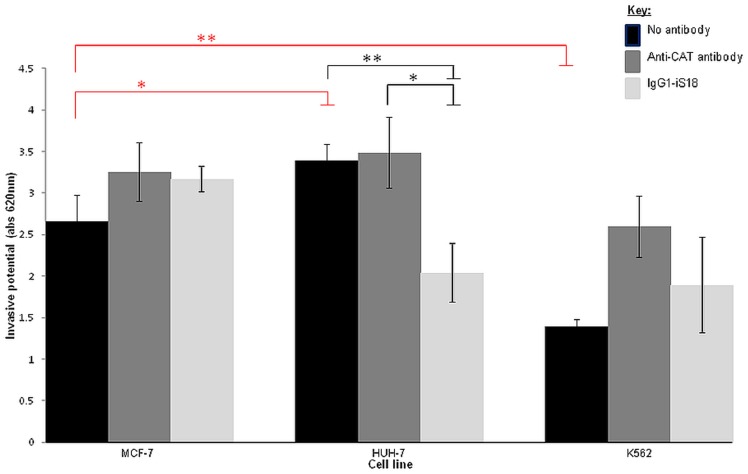
Effect of anti-LRP/LR specific antibody IgG1-iS18 on the invasion through the ECM-like Matrigel™ by liver cancer (HUH-7) and leukaemia (K562) cells. p-values indicated in red (*p = 0.0485; **p = 0.0053) represent the changes in invasive potential of cell lines in comparison to the MCF-7 control, whilst p-values shown in black (*p = 0.0214; **p = 0.0091) are indicative of the effect of appropriate antibodies on the invasive potential of the cell lines. Upon administration of IgG1-iS18 to the HUH-7 liver cancer cells, a reduction of approximately 39.75% was observed regarding the invasive potential of the cells. Data is representative of experiments carried out in triplicate and repeated at least three times.

As shown in Fig.7, liver cancer (HUH-7) cells are significantly more invasive in comparison to the poorly-invasive breast cancer (MCF-7) control cell line, whilst leukaemia (K562) cells showed a significantly lower invasive potential compared to the control. Moreover, IgG1-iS18 successfully hampered the invasive potential of liver cancer (HUH-7) cells whilst no significant result was observed for the leukaemia cells. As expected, the anti-CAT antibody control did not significantly affect the invasive potential of the tumorigenic cell lines.

## Discussion

Several studies have revealed that, on the cell surface, various tumorigenic cell lines exhibit an overexpression of the 37 kDa/67 kDa LRP/LR, therefore suggesting that the LRP/LR-laminin-1 interaction may be pivotal for cancer cells to undergo metastasis[21]. It may therefore be useful to inhibit this interaction as a means of hampering adhesion and invasion – two events found to be crucial to the occurrence of the process of metastasis. A study conducted by Zuber *et al* has demonstrated that the IgG1-iS18 antibody is highly specific for LRP/LR[18]. Furthermore, recent research has shown that anti-LRP/LR specific antibody IgG1-iS18 significantly impedes the adhesive and invasive potential of cervical[4], lung[4], prostate[4], colon[4], breast[20] and oesophageal[20] cancer cells. The present study investigated the role of LRP/LR in the adhesion and invasion of liver cancer (HUH-7) as well as leukaemia (K562) cells, and aimed to establish whether application of anti-LRP/LR specific antibody IgG1-iS18 significantly reduces the adhesive and invasive potential of these two tumorigenic cell lines.

Initially, confocal microscopy revealed that all three tumorigenic cell lines indeed display LRP/LR on their cell surface (Fig. 1a). However, this technique is limited by the fact that it is not quantitative and further analysis was required in order to establish the levels at which LRP/LR is displayed on the cell surface of these tumorigenic cell lines.

Significantly high percentages (>87%) of all three tumorigenic cell lines, namely HUH-7, K562, and MCF-7 (poorly-invasive breast cancer control) cells displayed LRP/LR on their cell surface (Fig.2). Furthermore, it was observed by analysis of differences in median fluorescence intensities that, in comparison to the MCF-7 control cell line, liver cancer cells (HUH-7) revealed significantly higher and leukaemia cells (K562) revealed significantly lower cell surface LRP/LR levels (Fig.3). As previously stated, LRP/LR plays essential roles in adhesion, invasion, proliferation and migration of cells[9]. Seeing that the HUH-7 cell line is known to be invasive and the K562 is understood to be a suspension cell line (ATCC), the cell surface levels of LRP/LR that have been observed may be correlating with the invasive potential of these cell lines.

Additionally, total LRP/LR levels were analysed by Western blotting in order to account for LRP/LR in the nucleus and cytosol of the tumorigenic cell lines. Western blot analysis (Fig.4) confirmed that all three tumorigenic cell lines express the 37 kDa LRP, however densitometry analysis of these blots (Fig.5) revealed that both the invasive and poorly-invasive cell lines show similar levels of total LRP and no significant differences were observed. It is noteworthy to state that in the nucleus and cytosol, LRP/LR serves particularly to maintain nuclear structures and facilitate translational processes, respectively[8]. Hence, even though total LRP levels do not differ significantly between the invasive and poorly-invasive tumorigenic cell lines, the cell surface LRP/LR levels are of importance to the occurrence of adhesion and invasion in the invasive and poorly-invasive tumorigenic cell lines. The current study revealed differences in only cell surface levels of LRP/LR between the three tumorigenic cell lines, and this is in agreement with results obtained in previously published research [4]. However, another study contradicted these results by showing that tumorigenic cell lines differed only in total LRP/LR levels[20]. These discrepancies in the latter-mentioned study could be owing to the fact that those cancerous cells may require enhanced protein synthesis in order to carry out metastatic processes, hence increased total LRP/LR levels were observed rather than increased cell surface LRP/LR levels.

The results obtained in the present study suggests that anti-LRP/LR specific antibody IgG1-iS18 caused a significant reduction in the adhesive potential of metastatic liver cancer cells on laminin-1 and additionally hampered the invasive potential of this cancer type on the Matrigel. Figures 6 and 7 depict these significant decreases in the adhesive and invasive potential of metastatic liver cancer (HUH-7) cells upon administration of IgG1-iS18, respectively. It is noteworthy to state that leukaemia (K562) cells showed no significant reduction in the adhesive and invasive potential upon administration of IgG1-iS18. It may be suggested that variations in the adhesive and invasive potential of both HUH-7 and K562 cell lines may be attributed to variations in cell surface LRP/LR levels. Hence, lower cell surface LRP/LR levels observed for leukaemia cells (Fig.3) could be held accountable for the lower adhesive and invasive capacity observed in this cell line, and vice versa for HUH-7 cells which exhibited high cell surface LRP/LR levels (Fig.3) and resulted in the increased adhesive and invasive capacity of this cell line. The significant reduction in the invasive potential of metastatic liver cancer cells after administration of anti-LRP/LR specific antibody IgG1-iS18 may be attributed to the inhibition of adhesion (Fig.6) by the IgG1-iS18 antibody, as adhesion is understood to be a pre-requisite for the occurrence of invasion during the induction of metastasis[20].

On the contrary, the K562 leukaemia cells were observed to be more adherent (Fig.6) but less invasive (Fig.7) than the poorly-invasive MCF-7 control cell line. This observation could be due to K562 cells expressing tissue inhibitors of metalloproteinases (TIMPs) and these TIMPs could be resulting in the inhibition of type IV collagenase activity[22], thereby preventing degradation of type IV collagen in the Matrigel and preventing K562 cells from undergoing invasion *in vitro*.

Analysis of the correlation between cell surface levels of LRP/LR with the adhesive and invasive potential of liver cancer (HUH-7) and leukaemia (K562) cells, resulted in considerably high correlation coefficients (Table 2). This signifies a positive and directly proportional relationship between the two parameters. Hence, this confirms that adhesion is a pre-requisite for invasion to occur, as seen by the high correlation coefficients obtained for adhesive to invasive potential for both experimental cell lines. Furthermore, the high correlation coefficients obtained for cell surface LRP/LR levels to the adhesive and invasive potential of the cell lines suggests that the aggressiveness of these two cancer types is enhanced by high levels of cell surface LRP/LR, which is consistent with results obtained by previous studies [4], [20]. It is important to note that only cell surface levels of LRP/LR were considered in the calculations of Pearson's correlation coefficients since no significant difference was observed in total LRP/LR levels between the three tumorigenic cell lines (Fig.5).

**Table 2:** Pearson's correlation coefficients between cell surface LRP/LR levels and the adhesive and invasive potential of liver cancer (HUH-7) and leukaemia (K562) cells.

Cell lines	LRP/LR levels to adhesive potential	LRP/LR levels to invasive potential	Adhesive to invasive potential
HUH-7	0.95	0.96	0.84
K562	0.91	0.99	0.88

In Table 2, the high Pearson's correlation coefficients observed between the adhesive and invasive potential for both cell lines ascertains that adhesion is indeed a mandatory step for the occurrence of invasion, where lower adhesive potential in leukaemia cells subsequently resulted in a lower invasive potential as well. This finding is in line with that of previously published literature which shows that the LRP/LR-laminin-1 interaction is pivotal for adhesion as well as secretion of enzymes that degrade the basement membrane and therefore promote invasion through the basement membrane[23].

Several studies have suggested that anti-LRP/LR tools such as pentosan sulphates and monoclonal antibodies directed towards the laminin receptor have shown the potential to significantly impede the adhesive and invasive potential of selected cancers such as laryngeal carcinoma cells as well as human fibrosarcoma cells through the interruption of the LRP/LR-laminin-1 interaction [18], [24].

Overall, it is strongly suggested that proteolytic cleavage of the basal lamina and subsequently the process of invasion is significantly enhanced through the LRP/LR-laminin-1 interaction[23]. Furthermore, anti-LRP/LR specific antibody IgG1-iS18 has been shown in the present study to significantly impact on the behaviour of metastatic liver cancer cells at the critical stages of adhesion and invasion *in vitro*, thereby suggesting the use of the antibody as an alternative therapeutic tool in the treatment of metastatic liver cancer.

Due to different cancer types exhibiting different behavioural characteristics, it cannot be assumed that anti-LRP/LR specific antibody IgG1-iS18 will have the same effect on all cancer types. Hence, the results of the current study will assist in providing the scientific community with novel aspects regarding the use of the antibody as a possible therapeutic tool for metastatic liver cancer. Furthermore, studies concerning appropriate delivery systems for the IgG1-iS18 antibody need to be conducted since LRP/LR plays critical roles in several essential physiological processes and the targeting of LRP/LR specifically in tumorigenic cells may prove to be difficult. Successful animal trials may indeed deem the antibody as a potential therapeutic tool for metastatic cancers.

## Supporting Information

Figure S1 **Quantification of liver cancer (HUH-7) and leukaemia (K562) cells within a population which display the CAT protein on their cell surface.** The first peak in graphs A,C and E represents cells labelled with APC-coupled secondary antibody only, whilst the second peak indicates cells that are labelled with both anti-CAT antibody as well as the secondary antibody. The unstained control is included in graphs B, D and F to confirm that the secondary antibody does not significantly bind non-specifically. Experiments were performed in triplicate and repeated at least three times with 20000 cells counted per sample.
(TIF)

Figure S2 **Quantification of cell surface CAT protein levels on liver cancer (HUH-7) and leukaemia (K562) cells by flow cytometric analysis.** Cells were labelled with anti-CAT antibody and APC-coupled secondary antibody. An analysis was performed on 20000 cells per sample across all three cell lines. The median fluorescence intensities of the samples labelled with both anti-CAT antibody and the secondary antibody were used as an indicator of CAT expression on the cell surface (with the unstained control being taken into account). The MFI value for the MCF-7 cell line wasset to 100%. Experiments were carried out in triplicate and repeated at least three times. N.S: p>0.05.
(TIF)

Figure S3 **Flow cytometric gating of MCF-7 (poorly-invasive breast cancer), HUH-7 (liver cancer) and K562 (leukaemia) cell samples.** Cells were gated to exclude debris and aggregated cells from the analysis. R1 and R3 indicate the gated cell population.
(TIF)
