# Supplementary figures and images for: Anti-LRP/LR Specific Antibody IgG1-iS18 Impedes Adhesion and Invasion of Liver Cancer Cells
Source: PLoS One. 2014 May 5;9(5):e96268. doi: 10.1371/journal.pone.0096268 (PMC4010454; doi:10.1371/journal.pone.0096268)

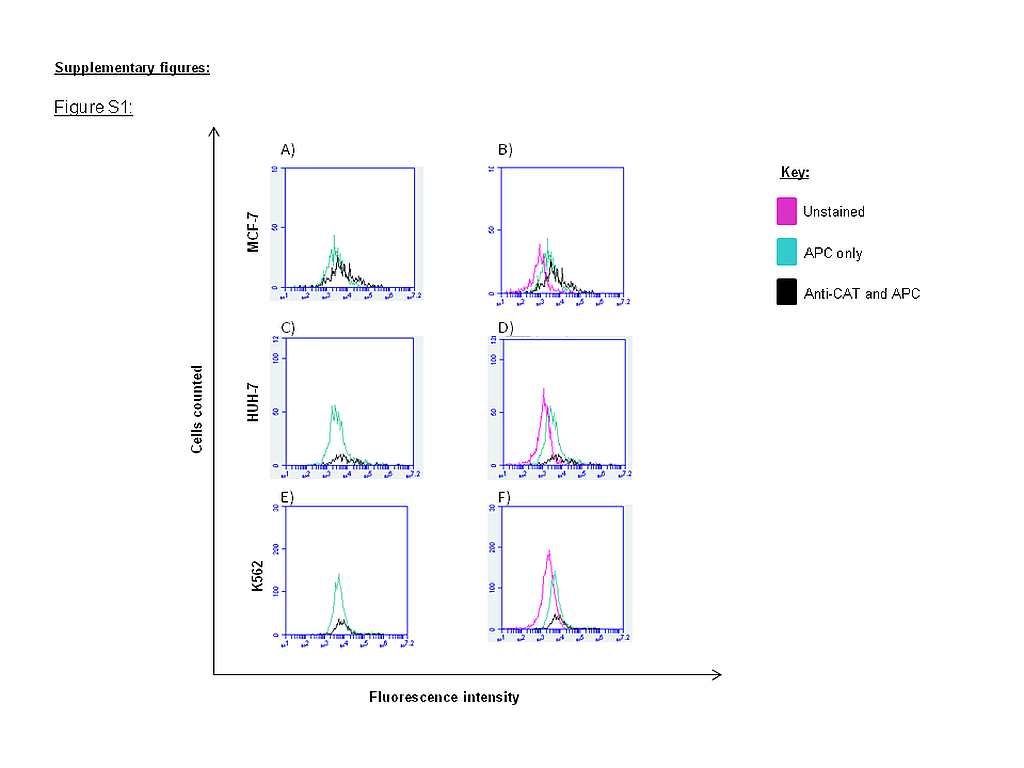

Supplement: Figure S1 — Quantification of liver cancer (HUH-7) and leukaemia (K562) cells within a population which display the CAT protein on their cell surface. The first peak in graphs A,C and E represents cells labelled with APC-coupled secondary antibody only, whilst the second peak indicates cells that are labelled with both anti-CAT antibody as well as the secondary antibody. The unstained control is included in graphs B, D and F to confirm that the secondary antibody does not significantly bind non-specifically. Experiments were performed in triplicate and repeated at least three times with 20000 cells counted per sample. (TIF) [file pone.0096268.s001.tif]

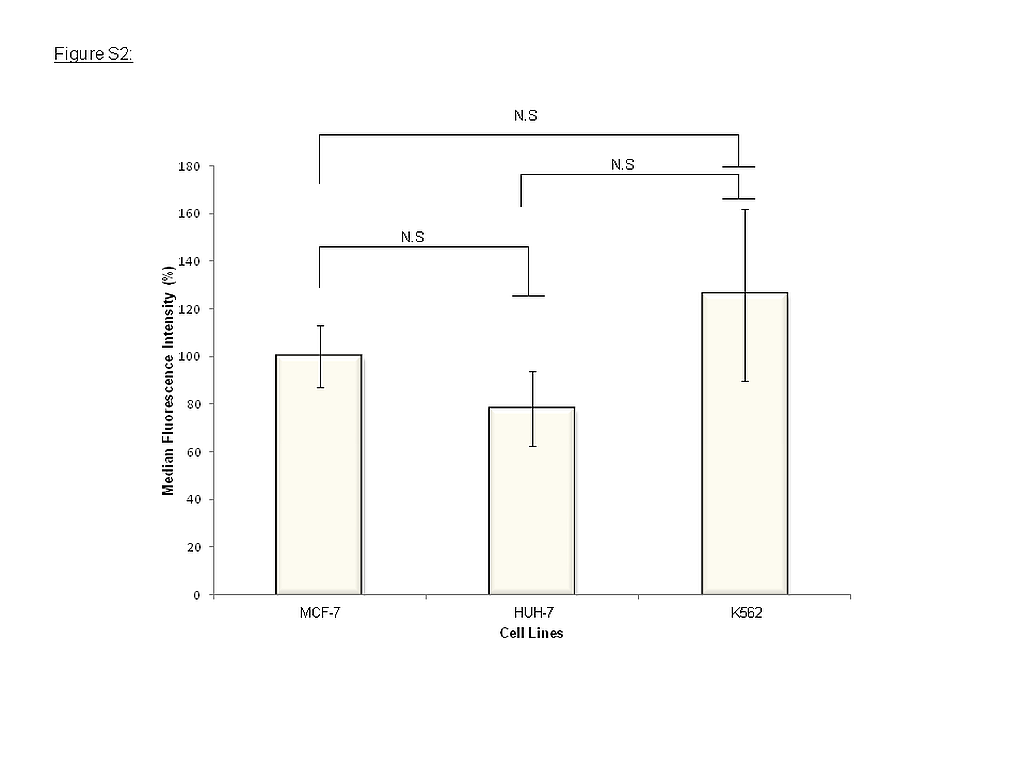

Supplement: Figure S2 — Quantification of cell surface CAT protein levels on liver cancer (HUH-7) and leukaemia (K562) cells by flow cytometric analysis. Cells were labelled with anti-CAT antibody and APC-coupled secondary antibody. An analysis was performed on 20000 cells per sample across all three cell lines. The median fluorescence intensities of the samples labelled with both anti-CAT antibody and the secondary antibody were used as an indicator of CAT expression on the cell surface (with the unstained control being taken into account). The MFI value for the MCF-7 cell line wasset to 100%. Experiments were carried out in triplicate and repeated at least three times. N.S: p>0.05. (TIF) [file pone.0096268.s002.tif]

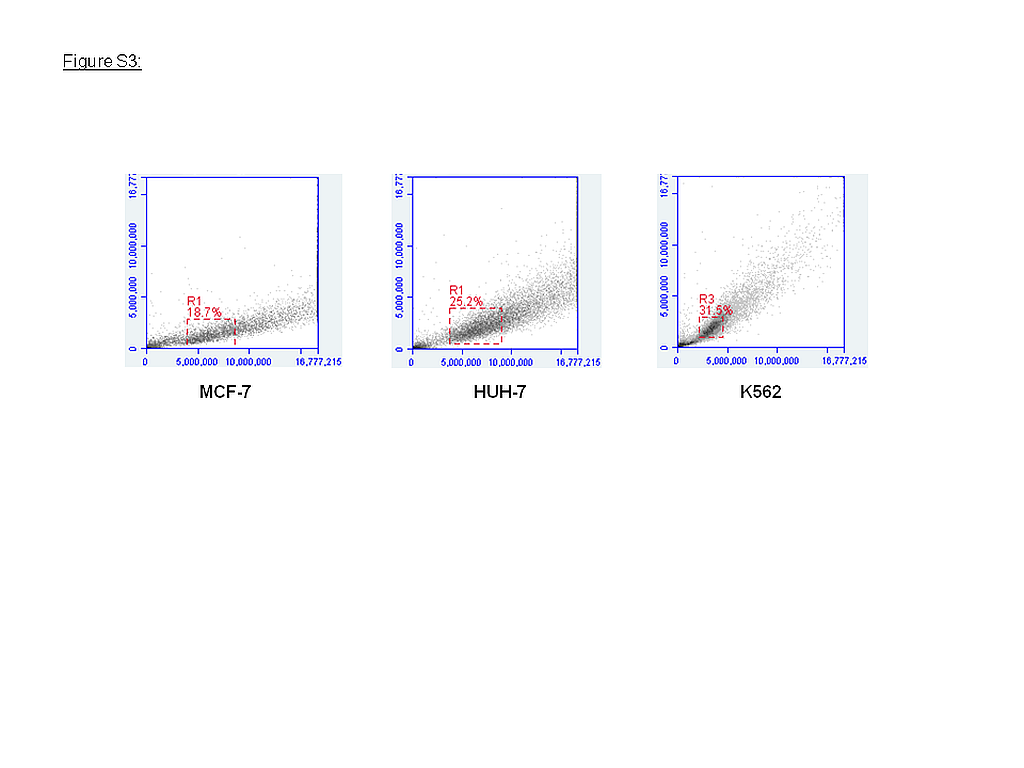

Supplement: Figure S3 — Flow cytometric gating of MCF-7 (poorly-invasive breast cancer), HUH-7 (liver cancer) and K562 (leukaemia) cell samples. Cells were gated to exclude debris and aggregated cells from the analysis. R1 and R3 indicate the gated cell population. (TIF) [file pone.0096268.s003.tif]
